# Editorial: GnRH system contribution in cancer regulation and clinical treatment

**DOI:** 10.3389/fendo.2024.1375416

**Published:** 2024-02-08

**Authors:** Euclides Avila, Tatiana Fiordelisio, Marco Allán Pérez-Solis, Guadalupe Maya-Núñez

**Affiliations:** ^1^ Departamento de Biología de la Reproducción Dr. Carlos Gual Castro, Instituto Nacional de Ciencias Médicas y Nutrición Salvador Zubirán, Mexico City, Mexico; ^2^ Laboratorio de Neuroendocrinología Comparada, Laboratorio Nacional de Soluciones Biomiméticas para Diagnóstico y Terapia, Biología, Facultad de Ciencias, Universidad Nacional Autónoma de México, Ciudad Universitaria, Mexico City, Mexico; ^3^ Unidad de Investigación Médica en Medicina Reproductiva, Coordinación de Investigación en Salud, Instituto Mexicano del Seguro Social, Mexico City, Mexico

**Keywords:** GnRH (gonadotropin-releasing hormone), GnRH receptor, cancer regulation, targeted-therapy, antiproliferative activity

Gonadotropin releasing hormone (GnRH) acts as a key mediator in regulating the hypothalamic-pituitary-gonadal (HPG) axis. Synthesized within the median hypothalamic neurons, GnRH is released into the hypothalamic-hypophysial-portal circulation, and interacts with its receptor (GnRHR), on gonadotropin cells in the anterior pituitary. This interaction modulates gonadotropin cell activity, leading to the synthesis and release of luteinizing hormone (LH), and follicle-stimulating hormone (FSH). Released into the systemic circulation, LH and FSH are pivotal for promoting gonadal growth, gametogenesis, and sex steroids production. Gonadal steroids, in a reciprocal manner, exert positive or negative feedback responses at different levels of the HPG axis. It is important to note that GnRHR expression is not limited to the anterior pituitary; it extends to tissues such as the ovary, placenta, uterus, and brain.

The involvement of GnRHR in carcinogenic processes has gained significance, with their identification on the cell membrane of malignant cells from different types of cancer, including endometrial, ovarian, prostate, and breast cancers. This presence extends to non-reproductive tumors like liver and lung. In breast cancer, GnRHR is detected in nearly 50% of samples, with higher prevalence in triple-negative breast cancer and invasive ductal carcinomas (around 64%). However, the expression of this receptor in normal breast tissues remains elusive. Reports indicate that GnRHR expressed in cancer cells directly influences cancer progression, and GnRH analogs exhibit direct anti-cancer actions by modulating Gα subunits. These GnRH analogs induce apoptosis in prostate cancer cells, presumably via Gαi coupling. The GnRH agonists can antagonize growth factor-induced mitogenic signaling in certain ovarian and endometrial cancer cell lines, suppressing proliferation. Mechanisms underlying the antiproliferative actions of GnRH or its analogs in cancer cells include ERK-mediated dephosphorylation of cell cycle regulator retinoblastoma protein, inhibition of phosphatidylinositol kinase activity, and down-regulation of telomerase reverse transcriptase and vascular permeability factor expression. In OVCAR-3 and MCF-7 cells, ERα, but not ERβ, represses the GnRHR promoter, suggesting a predominant role of ERα in controlling cell proliferation in ovarian and breast cancers, where both receptor subtypes are expressed. Despite considerable information about the role of GnRHR in regulating proliferation and metastasis in various cancer models, the implications for potential treatment require further investigation. This Research Topic emphasizes the importance of the GnRHR in cancer regulation. The manuscript by Garrido et al. reviews recent advances in GnRH-based drug delivery to enhance chemotherapeutic drug potency in ovarian, breast, and prostate cancer, with special emphasis on GnRH-targeted nanoparticles. The normal regulation of the HPG axis is complex; AXL, a tyrosine kinase receptor, and its ligand Gas6 play a role in this system. AXL/Gas6 signaling is involved in the correct migration of GnRH neurons, and many cancer cells exhibit AXL-dependent PI3K/Akt signaling, contributing to proliferation, metastasis, invasion, and drug resistance. In addition AXL signaling suppresses GnRH expression in immortalized non-migratory neurons. The review by Mohammadzadeh and Amberg details research on AXL/Gas6 signaling mechanisms in reproductive health and disease. Adding complexity, over 20 primary structures of GnRH and its receptors have been identified across species. Cho-Clark et al. describe a GnRH metabolite, GnRH-(1-5), with a response contrary to GnRH, especially in endometrial cancer, through the GPR101 receptor a truncated form of a full-length protein like GnRHR. Another form of GnRH, present also in mammals is GnRH2 and binds to GnRHR2. Desaulniers and White present an extensive review of the role of GnRH2 and GnRHR2 in reproductive cancers, suggesting GnRH2 as an emerging target for treating these cancers.

The present overview aims to stimulate interest in understanding the involvement of GnRH signaling in cancer regulation and its potential application as an emerging pharmacological targeted therapy.

## Author contributions

EA: Investigation, Supervision, Writing – review & editing, Conceptualization. TF: Investigation, Supervision, Writing – review & editing, Conceptualization. MP: Investigation, Supervision, Writing – review & editing, Conceptualization. GM: Conceptualization, Investigation, Supervision, Writing – original draft, Writing – review & editing.

